# Advanced Hepatocellular Carcinoma with Subtotal Occlusion of the Inferior Vena Cava and a Right Atrial Mass

**DOI:** 10.1155/2013/489373

**Published:** 2013-04-11

**Authors:** Christian Steinberg, Suzanne Boudreau, Felix Leveille, Marc Lamothe, Patrick Chagnon, Isabelle Boulais

**Affiliations:** ^1^Department of Cardiology, Quebec Heart and Lung Institute, Laval University, 2725 Chemin Sainte-Foy, Quebec, QC, Canada G1V 4G5; ^2^Department of Pathology, Hôtel-Dieu d'Arthabaska, 5 rue des Hospitalières Victoriaville, QC, Canada G6P 6N2; ^3^Department of Nuclear Medicine, Hôtel-Dieu d'Arthabaska, 5 rue des Hospitalières Victoriaville, QC, Canada G6P 6N2; ^4^Department of Internal Medicine, Hôtel-Dieu d'Arthabaska, 5 rue des Hospitalières Victoriaville, QC, Canada G6P 6N2

## Abstract

Hepatocellular carcinoma usually metastasizes to regional lymph nodes, lung, and bones but can rarely invade the inferior vena cava with intravascular extension to the right atrium. We present the case of a 75-year-old man who was admitted for generalized oedema and was found to have advanced HCC with invasion of the inferior vena cava and endovascular extension to the right atrium. In contrast to the great majority of hepatocellular carcinoma, which usually develops on the basis of liver cirrhosis due to identifiable risk factors, none of those factors were present in our patient.

## 1. Introduction

Primary hepatocellular carcinoma (HCC) is a quite uncommon tumor in North America and Western Europe but is the fifth most common cancer worldwide and the third leading cause of cancer-related death [1, 2]. Most cases of HCC occur in patients with chronic liver disease or preexisting liver cirrhosis. Common causes for liver cirrhosis are chronic alcoholic liver disease or chronic viral hepatitis due to hepatitis B virus or hepatitis C virus infection [2]. Other risk factors for the development of HCC are metabolic diseases like hemochromatosis or alpha1-antitrypsin deficiency, autoimmune liver diseases (autoimmune hepatitis, primary biliary cirrhosis), and aflatoxin exposition [1, 3–5]. The incidence of HCC shows striking variations between different geographic regions and among different racial and ethnic background within the same country, suggesting a crucial role of genetic and environmental factors in the pathogenesis of HCC [6, 7]. HCC is an aggressive tumor and can show extensive metastazation. It usually metastasizes to regional lymph nodes, lung, or bone but sometimes shows invasion of major blood vessels with endovascular extension [8, 9]. In this report, we present the rare case of an advanced hepatocellular carcinoma with invasion of the inferior vena cava and intravascular extension to the right atrium in a patient without any preexisting liver disease.

## 2. Case Report

A 75-year-old Caucasian man presented to the emergency room of our hospital for dyspnea and new onset generalized oedema rapidly progressing over one week. The patient was known for stable coronary artery disease, paroxysmal atrial fibrillation with oral anticoagulation, chronic obstructive bronchitis, hypertension, and dyslipidemia. He had stopped smoking 3 years before, had no history of alcoholism, and had never taken illegal drugs. 

Vital signs at presentation were stable. The patient was afebrile and not in respiratory distress. Physical examination revealed generalized oedema associated with ascites and hepatomegaly. The jugular veins were not distended, but there was a strong clinical suspicion of a right-sided pleural effusion. An initial chest X-ray confirmed an important right pleural effusion and a 1 cm sized nodule in the right lower lobe (not shown). Results of laboratory tests are shown in Table 1. Most strikingly, there was a new onset perturbation of liver markers. 

**Figure 1:**
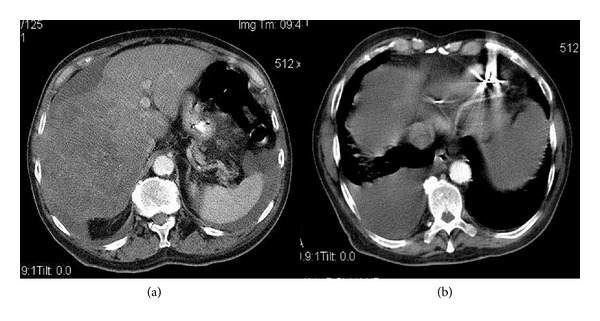
Contrast enhanced CT scan of thorax, abdomen, and pelvis.

**Figure 2:**
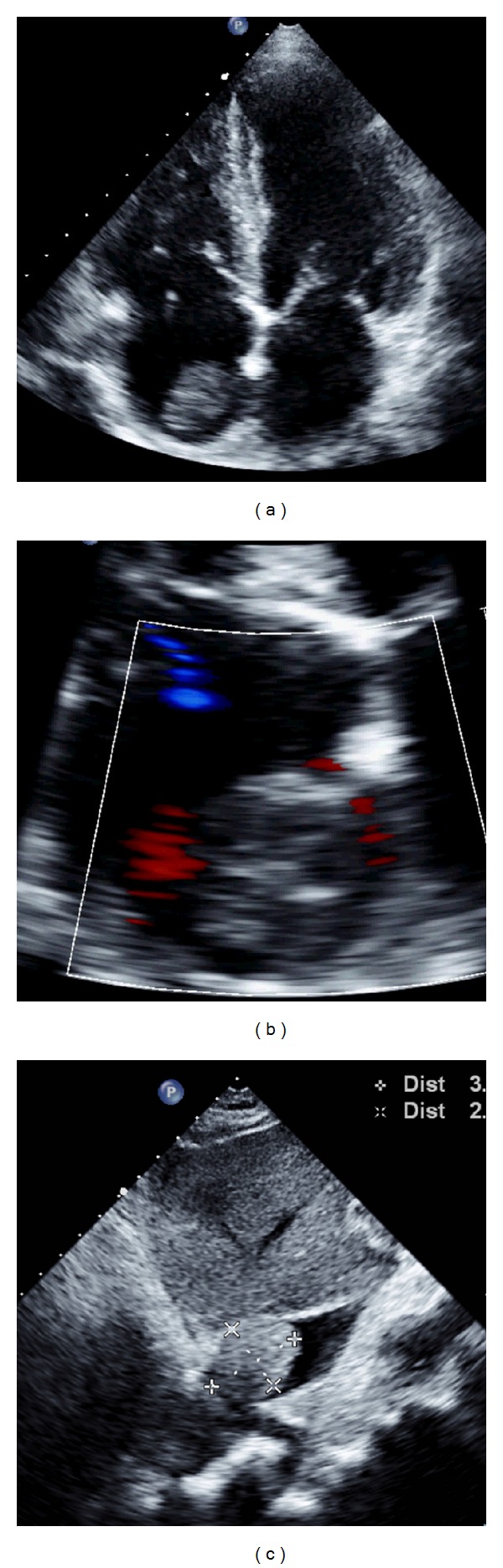
Transthoracic echocardiography.

A contrast enhanced CT scan of the chest, abdomen, and the pelvis was performed. The abdominal CT scan showed a very heterogeneous liver. Except for segments 2 and 3, the whole liver contained multiple ill-defined and partially confluent hypodensities of different size suggesting an advanced neoplastic process (Figure 1(a)). There was also a doubt of a hypodense lesion inside the inferior vena cava (Figure 1(b)). The hepatic lesions were associated with a moderate quantity of ascites but no splenomegaly (Figure 1(a). The chest study confirmed the presence of a large right-sided pleural effusion and a 1 cm sized nodule in the anterior part of the right lower lung lobe (not shown). On transthoracic echocardiography, a well-defined, immobile oval mass with a smooth surface and a size of 23 mm × 30 mm was noted in the right atrium (Figure 2(a)). The mass was not adherent to the interatrial septum (Figure 2(b)). The mass extended to the inferior vena cava where it reached a size of 34 mm × 25 mm, creating a subtotal occlusion with a pressure gradient of 13 mmHg between the inferior vena cava and the right atrium (Figure 2(c)). The occluded inferior vena cava showed no respiratory compliance and had a diameter of 21 mm.

**Figure 3:**
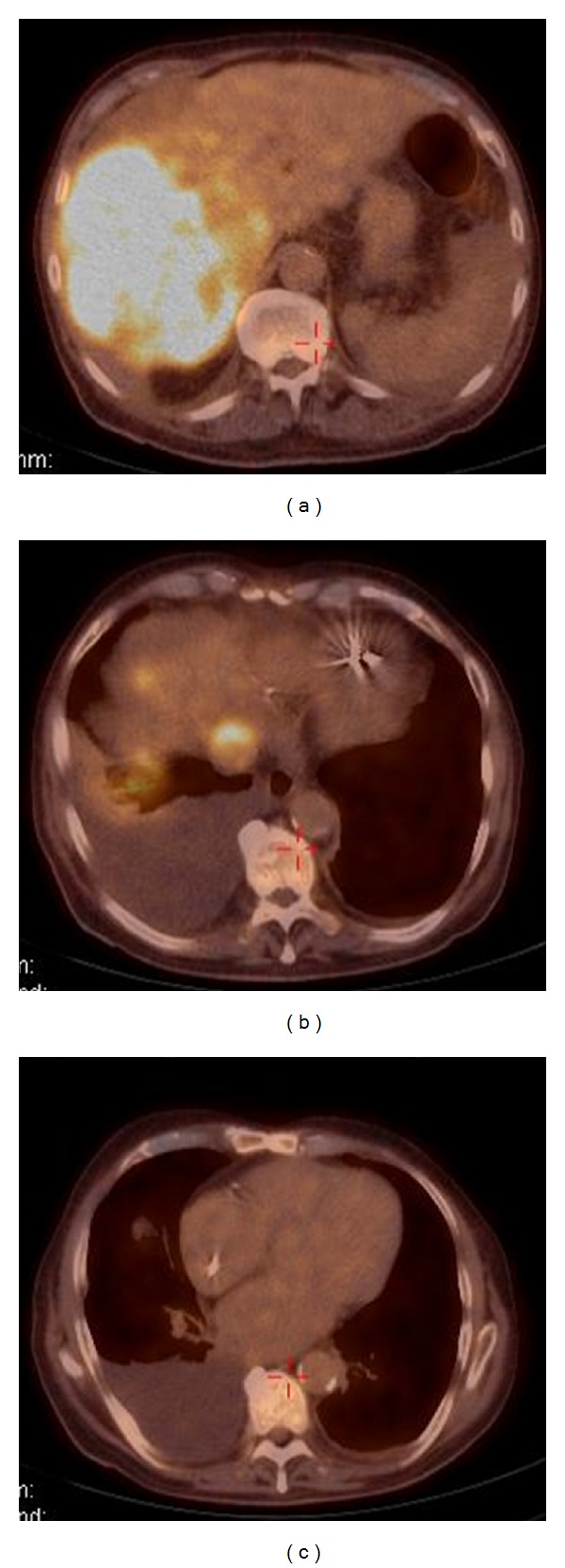
Positron emission tomography-CT.

A complete colonoscopy was negative for a neoplastic lesion, so liver biopsy under CT guidance was performed to establish a histological diagnosis. To complete the tumor staging and further characterize the endovascular lesion of the inferior vena cava and right atrium, a positron emission tomography-CT (PET-CT) was performed. On the PET imagery, there was a strong hypermetabolic zone in the right liver lobe covering an area of 13 cm × 14 cm × 13 cm (maximal normalized capture index 8.9) (Figure 3(a)). This hypermetabolic zone corresponded to the hepatic lesions seen on the CT scan and extended inside the inferior vena cava over a distance of 3.5 cm (Figure 3(b)). The hypermetabolic zone stopped at the junction of the inferior vena cava with the right atrium. No abnormal hypermetabolism was noted inside the heart (Figure 3(c)). The lung nodule seen on the CT scan was also hypermetabolic with a capture index of 2.3 suggesting a metastasis (not shown).

**Figure 4:**
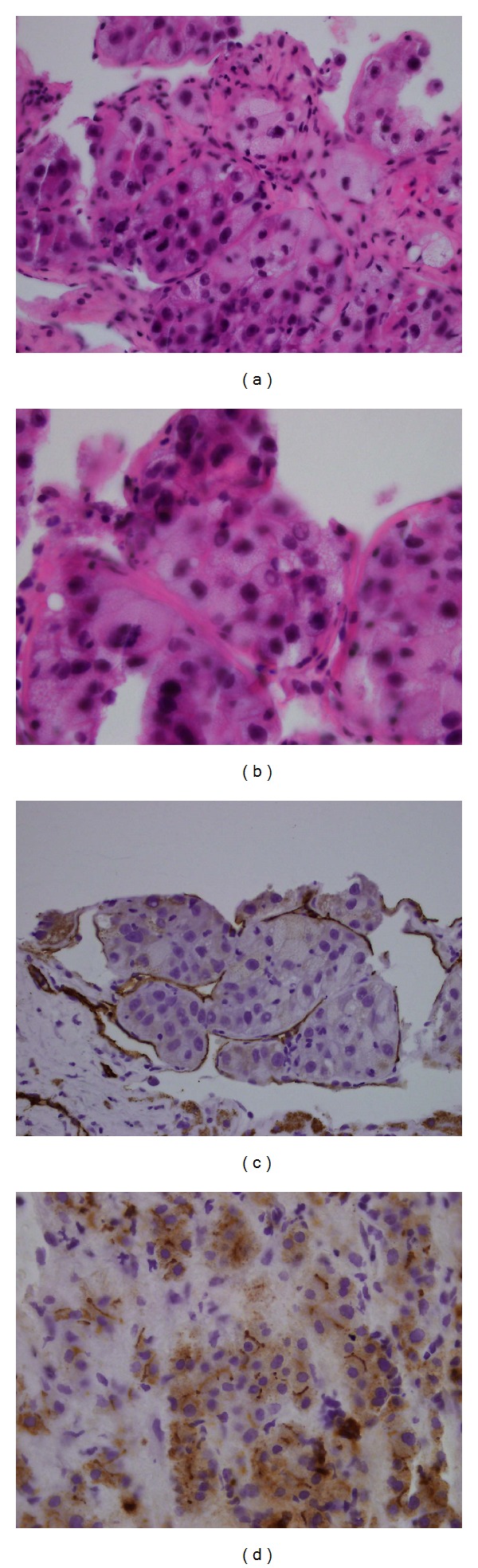
Liver biopsy. Hematoxylin and eosin staining (a) and (b). Immunostaining for CD34 (c) and for carcinoembryonic antigen (CEA) (d).

The histological examination of the liver biopsy demonstrated a hepatocellular carcinoma with a well- and a poorly-differentiated component (Figure 4). There were no microscopical signs of liver cirrhosis on the specimen. The serum level of alpha-fetoprotein was 270 000 ng/mL. Taken together, these results established the final diagnosis of a stage IV locally advanced hepatocellular carcinoma with endovascular extension and a single pulmonary metastasis. It remains unclear if the metabolic inactive part of the endocaval and intra-atrial mass represented a superimposed thrombosis or necrotic tumor tissue. 

Laboratory tests to screen underlying risk factors of HCC were performed. Virus serology for hepatitis B, hepatitis C, and human immunodeficiency virus was negative. The serum ferritin level was 398 *μ*g/L, and the serum iron saturation was at 12%. The electrophoresis of serum proteins was normal eliminating alpha1-antitrypsin deficiency.

Confronted with the diagnosis, the patient did not desire further treatment and was orientated to palliative care.

## 3. Discussion

Although HCC usually metastasizes to regional lymph nodes, lung, or bones, primary liver cancer has also the propensity to invade major local blood vessels with intravascular extension [8, 9]. Based on autopsy series, invasion of the inferior vena cava has been described in up to 9%–26% and intravascular extension to the right atrium in 2.4%–6.3% of cases of HCC [10–12]. Virtually all reported cases of HCC with intracaval invasion had preexisting liver cirrhosis and/or at least one classical risk factor for HCC [8, 9, 11]. Our patient differs from the known literature in that he had neither any classical risk factors for HCC nor histological signs of underlying cryptogenic cirrhosis. 

Patients with tumor invasion of the inferior vena cava and/or intravascular extension to the heart have a very poor outcome. Typical complications of intravascular tumor extension lead to secondary Budd-Chiari syndrome, right heart insufficiency, or massive pulmonary embolism secondary to detached tumor tissue or superimposed thrombotic material [13]. Local surgical and nonsurgical approaches as well as systemic therapy with antiangiogenic agents have been described for HCC patients with caval invasion. Very few patients are candidates for local surgery because of the high perioperative morbidity and mortality of those high-risk interventions, and clinical experience is limited to occasional cases or small series [14, 15]. Nonsurgical local treatments like transarterial chemoembolization or local radiotherapy are only moderately effective and are also associated with important morbidity [16, 17]. While HCC is little responsive to classical cytotoxic chemotherapy, the focus of systemic therapy has shifted to immunomodulatory molecules [18]. Among them, Sorafenib, an oral multikinase inhibitor, has shown to prolong median survival and time to radiologic progression and has now become standard treatment for advanced HCC [19]. Another option is Thalidomide, which is an oral systemic inhibitor of angiogenesis. Treatment with Thalidomide for advanced HCC has been in the focus of interest over the last years, but clinical data show only limited activity and are inconclusive [20–22]. So far, only one study describes the use of Thalidomide in HCC patients with intra-atrial tumor extension. In the study of Chang et al., three patients with advanced HCC and inferior vena cava/right atrium tumor thrombi were assigned to Thalidomide. Two of the patients responded to Thalidomide with a survival of 15 months, whereas the third patient had symptomatic palliation [8]. 

## 4. Conclusion

Most cases of HCC develop on the basis of preexisting chronic liver disease with identifiable risk factors. Invasion of inferior vena cava represents a rare but catastrophic complication. We report the rare case of a patient with advanced HCC without any classical risk factors or underlying cirrhosis who presented with generalized oedema as a consequence of tumor invasion of the inferior vena cava with endovascular extension to the right atrium.

## Figures and Tables

**Table 1:** Laboratory results on admission.

Red blood cells	6.2 × 10^12^/L
Hemoglobin	160 g/L
Hematocrit	0.50
INR	5.10
AST	21 U/L
ALT	91 U/L
Bilirubin (total)	20 *μ*mol/L
Alkaline phosphatase	164 U/L
Albumin	30 g/L
